# Efficient Neural Networks on the Edge with FPGAs by Optimizing an Adaptive Activation Function

**DOI:** 10.3390/s24061829

**Published:** 2024-03-13

**Authors:** Yiyue Jiang, Andrius Vaicaitis, John Dooley, Miriam Leeser

**Affiliations:** 1 Department of Electrical and Computer Engineering, Northeastern University, Boston, MA 02115, USA; yyjiang@coe.neu.edu; 2 Department of Electronic Engineering, Maynooth University, W23 F2H6 Maynooth, Ireland; andrius.vaicaitis.2016@mumail.ie (A.V.); john.dooley@mu.ie (J.D.)

**Keywords:** adaptive activation function (AAF), neural network, FPGA, deep learning, digital predistortion

## Abstract

The implementation of neural networks (NNs) on edge devices enables local processing of wireless data, but faces challenges such as high computational complexity and memory requirements when deep neural networks (DNNs) are used. Shallow neural networks customized for specific problems are more efficient, requiring fewer resources and resulting in a lower latency solution. An additional benefit of the smaller network size is that it is suitable for real-time processing on edge devices. The main concern with shallow neural networks is their accuracy performance compared to DNNs. In this paper, we demonstrate that a customized adaptive activation function (AAF) can meet the accuracy of a DNN. We designed an efficient FPGA implementation for a customized segmented spline curve neural network (SSCNN) structure to replace the traditional fixed activation function with an AAF. We compared our SSCNN with different neural network structures such as a real-valued time-delay neural network (RVTDNN), an augmented real-valued time-delay neural network (ARVTDNN), and deep neural networks with different parameters. Our proposed SSCNN implementation uses 40% fewer hardware resources and no block RAMs compared to the DNN with similar accuracy. We experimentally validated this computationally efficient and memory-saving FPGA implementation of the SSCNN for digital predistortion of radio-frequency (RF) power amplifiers using the AMD/Xilinx RFSoC ZCU111. The implemented solution uses less than 3% of the available resources. The solution also enables an increase of the clock frequency to 221.12 MHz, allowing the transmission of wide bandwidth signals.

## 1. Introduction

Wireless communications is an important area in edge computing and a growing application area for FPGAs. Machine learning is increasingly used in wireless applications, and as a result, compact and accurate implementations on FPGAs are in demand. For edge processing, consuming few resources and delivering low latency are important. This paper addresses the issue of machine learning applied to digital predistortion (DPD), an important application in wireless communications. Power amplifiers (PAs) are required for the transmission of radio-frequency signals, and DPD improves the linear operation of the transmitted signals, which is essential to avoid interference in radio communications.

Operators would like to run their PAs in the most power-efficient mode possible; however, these modes typically cause the PA to introduce more nonlinear distortion to the signal. DPD introduces nonlinear behavior, which is the inverse of that caused by the PA. FPGAs can be effective in implementing DPD, especially just before the RF frontend, where signals are transmitted and received. Coefficients for DPD need to be determined such that the particular implementation best linearizes the PAs under the current operating conditions, which may change over time. By implementing DPD at the edge, we can respond quickly to these current operating conditions. By using few resources on the FPGA, other frontend processing can be combined with DPD for wireless receivers and transmitters. We implemented DPD on an AMD/Xilinx RFSoC, which integrates the RF frontend with FPGA fabric to demonstrate the ability to quickly respond to the current operating conditions by changing the DPD coefficients with very little hardware overhead.

Neural networks have been applied to DPD, including real-valued time-delay neural networks (RVTDNNs) [1]. More recent neural network structures for DPD [2,3] have targeted enhanced dynamic range performance at the cost of increased network size, additional parameters, and long training times. Recently, CNNs have been applied to DPD [4,5,6]. However, these network structures have even more network parameters, resulting in greater computational complexity and longer training times. This is evident from observing the resource requirements for the FPGA implementation of CNNs for image classification [7,8] and more lightweight CNNs are developed to be fit into edge devices [9,10]. In general, network structures with fewer parameters require shorter training times and are more suitable for systems with resource limitations on edge devices; however, they must still be capable of delivering the required performance.

Most prior work on neural networks, for DPD and for other applications, makes use of fixed activation functions. Fixed activation functions are limited by their own nonlinear characteristics to mimic a nonlinear function and will cause accuracy loss during the training phase. To increase training accuracy, the common solution is to expand the size of the neural network model so that this will lead to more hardware cost during inference phase, which happens on edge devices in our discussion. For inference, nonlinear activation functions such as *tanh* and *sigmoid*, which are commonly used, are hard to implement directly, but are instead approximated using piecewise linear approximations or multiple-order-based approximations. These approximations cause the model to lose accuracy during the inference phase. At the same time, approximation-based hardware solutions may also increase costs in terms of resource usage while slowing the speed, as further elaborated in Section 3.1.

In this paper, we focus on an alternative approach, namely using an activation function that can be adapted during training. An adaptive activation function can better fit the nonlinear model during training compared to a fixed model, and this reduces the training loss [11,12]. This network structure and training strategy has been used in prior research [13,14,15]. However, there is little research that explores the hardware efficiency of implementing adaptive activation functions. This is the focus of this research.

In this work, we present a complexity-reduced and resource-saving, adaptive-activation-function-based segmented spline curve neural network (SSCNN) implementation for DPD and validate its operation on an AMD/Xilinx RFSoC platform. The SSCNN employs an adaptive activation function that can be better tailored for the objective of linearizing the PA while greatly reducing the hardware resources needed on the FPGA.

The contributions of this work are as follows:

- The first FPGA implementation of an adaptive-activation-function (AAF)-based neural network for regression.
- An adaptive activation function applied to digital predistortion and implemented in FPGA hardware.
- An optimized segmented spline curve layer that has been designed with the target hardware in mind to provide an implementation true to the mathematical basis of the segmented spline curve layer.
- The results show that the implementation of the AAF for DPD using the SSCNN is capable of effective linearization while consuming minimal resources.
- A thorough comparison of different neural network structures and their FPGA implementations that compares the performance and resources used. The comparison shows that the SSCNN has similar performance to a deep neural network (DNN) while using far fewer hardware resources.

The rest of this paper is organized as follows. Section 2 provides the background on DPD and introduces different neural network models. Section 3 discusses related research on activation functions, highlighting the shortcomings of fixed activation functions, and then, introduces the adaptive activation function, specifically the segmented spline activation function, presented in this paper. In Section 4, we describe our hardware design and implementation. Specifically, we analyze the segmented spline activation function mathematically in Section 4.1 and describe how each block is designed based on this analysis (Section 4.4). Throughout the section, we describe improvements to each block to make the whole system more efficient.

The results are presented in Section 5, including a comparison of different approaches with respect to performance and resource usage. Finally, we present conclusions and future work.

## 2. Background

In this section, we introduce the background regarding DPD with a focus on the commonly used neural network-based learning architectures. Then, we illustrate three different neural networks frequently used for DPD and introduce the adaptive-activation-function-based segmented spline curve neural network, which is the focus of this paper.

### 2.1. DPD

The role of digital predistortion in wireless communications is to place a predistorter before a power amplifier (PA) in order to linearize the amplifier’s nonlinear behavior before transmitting signals. Ideally, when cascading the predistorter and PA, the output of the PA becomes linear as the predistorter behaves as the inverse of the PA.

There is a version of the AMD/Xilinx RFSoC board, called the RFSoC DFE [16], that implements DPD in hardware as a separate block from the FPGA fabric. Such hardware blocks are only implemented by Xilinx after an in-depth study of the benefits of doing so. Hence, this illustrates the importance of DPD in wireless communications. The hardware implemented on the board is a memory polynomial structure [17] for single-input/single-output PAs. Our goal is to provide more adaptive DPD solutions by taking advantage of neural networks.

Neural networks (NNs) have an inherent parallel structure and are naturally suited to multiple PAs [18,19]. Many researchers are investigating NNs for DPD as NNs are more adaptable than the memory polynomial approach, due to the ability to take advantage of additional inputs such as the input signal magnitude or temperature. This is increasingly important for 5G and 6G scenarios. In this paper, we investigated efficient implementations of such DPD solutions that could be incorporated into hardware blocks in the future.

### 2.2. Direct Learning and Indirect Learning Architectures

**Figure 1:**
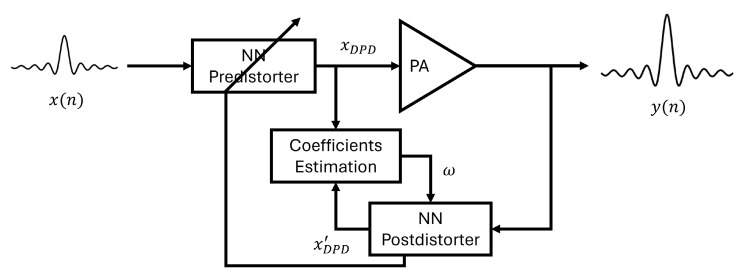
NN-based indirect learning architecture.

Learning the characteristics of the PA is key to generating the predistorter. Two commonly used architectures for identifying the coefficients of DPD are the direct learning architecture (DLA) and indirect learning architecture (ILA) [20,21,22]. The DLA first trains a PA model based on the PA’s input and output, then inverts this model to implement a predistorter. In the ILA, a postdistorter is trained as an inverse nonlinear PA model, and then, the coefficients are copied to the predistorter to achieve DPD. Because of its good performance and straightforward implementation, we adopted the ILA structure combined with different neural network models to estimate the parameters of DPD, as shown in Figure 1.

### 2.3. Real-Valued Time-Delay Neural Network

The RVTDNN [1,23] is one of the most commonly used neural network structures for DPD and PA behavioral modeling. The RVTDNN takes a complex signal’s in-phase (*I*) and quadrature (*Q*) components as two sets of real-valued inputs to the neural network and makes use of delay taps to incorporate memory effects in the PA model. One of its variants, the augmented real-valued time-delay neural network (ARVTDNN) [24], improves DPD’s performance by introducing envelope terms as additional inputs to the RVTDNN. These two commonly used neural network structures involve nonlinear activation functions, which increase computational complexity and cost when implemented in hardware.

### 2.4. Deep Neural Networks

Deep neural networks (DNNs) have also been utilized in DPD research [4,25]. DNNs, which are built with more than three layers, can sometimes offer superior accuracy compared to shallow neural networks due to their increased layer count. As demonstrated in [4], a DPD application using a DNN can outperform a shallow neural network, albeit at a significant hardware resource cost. Given that hardware implementations of DPD typically occur on edge devices and are, thus, constrained, DNNs may not be the best choice when solving DPD. The implementation of large and complex DNNs can be hindered by factors such as the requirements for the memory bandwidth and buffer size, which are needed for coefficient access during the inference phase, and are limited on edge devices. Recent efforts have been made to choose the best DNN structures for PA linearization [25], but these approaches still result in substantial hardware requirements to achieve the desired level of accuracy.

### 2.5. Segmented Spline Curve Neural Network

The SSCNN structure, an enhancement of the original RVTDNN structure, was first presented in [26]. This new structure significantly cuts down the number of necessary coefficients. It avoids the need for bias coefficients in the neurons of the hidden layer and incorporates the envelope terms directly at the output layer. Moreover, the SSCNN employs an adaptive activation function, discussed in Section 3, that can be tuned during the neural network training process. This gives the SSCNN an edge over other commonly used activation-function-based NNs for several reasons. Since the adaptive activation function substitutes a piecewise linear approximation in place of a nonlinear activation function, such as the hyperbolic tangent activation function, it provides better accuracy while reducing computational complexity in both the training and inference phases. In this study, we have designed an FPGA-based SSCNN structure based on a hardware-oriented, mathematical simplification that simplifies the calculation steps of the adaptive activation function. This design reduces hardware complexity and conserves resources in comparison to the commonly used nonlinear activation functions found in other structures, while maintaining accuracy.

**Figure 2:**
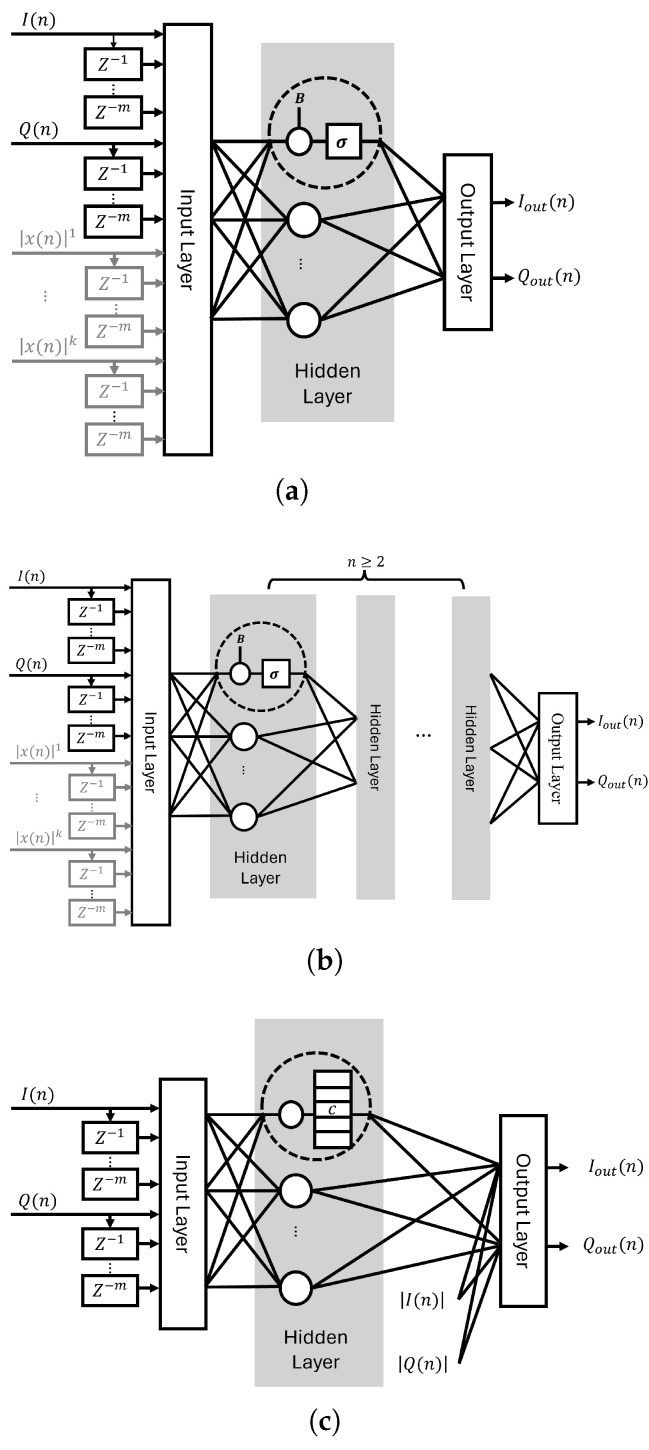
Different neural network structures. (**a**) (A)RVTDNN (grey). (**b**) DNN. (**c**) SSCNN.

In this paper, we compared our SSCNN implementation with other network structures including the RVTDNN, ARVTDNN, and DNNs, which utilize fully connected layers, specifically in the context of an FPGA implementation. The different neural network structures are shown in Figure 2.

## 3. Activation Functions

This section focuses on adaptive activation functions (AAFs). We first present related work on activation functions and discuss the problems introduced by fixed activation functions. We then present adaptive activation functions and, in particular, the segmented spline activation function, in more detail.

### 3.1. Related Work on Activation Functions

While a number of architectural optimizations for neural networks such as pruning have been the focus of studies for hardware implementations [27,28], activation functions that are used in neural networks have received far less attention. A major contribution of this research is the implementation of an adaptive activation function on an FPGA for a regression problem. In NNs, the same activation function is applied both during the training phase and the inference phase; hence, choosing an efficient activation function results in memory and computational savings for both phases. Currently, most neural networks that target regression problems use fixed activation functions such as *tanh* and *sigmoid*. The hardware implementation for the inference of these nonlinear functions relies on sub-functions such as $e^{x}$ and 1/x, which have high computational complexity and high latency. Most traditional activation functions are implemented based on piecewise linear and, in one case, piecewise second-order approximations. In [29,30], the *sigmoid* activation functions were broken down into linear segments with acceptable accuracy loss and were implemented using look-up tables (LUTs). One problem with these solutions is that using linear segments to mimic the *sigmoid* or *tanh* function makes the accuracy loss worse. The DCT interpolation-based *anh* function was implemented on an FPGA with minimal error, but required 21 look-up tables (LUTs) for an 8-bit output [31]. Si et al. (2020) [32] proposed a hardware-friendly D-ReLU activation function, but it is suitable for classification problems and not regression. Ngah et al. (2016) [33] designed a differential look-up table-based *sigmoid* function with limited accuracy. Gao et al. (2020) [34] designed a better *sigmoid* function on an FPGA for regression problems, but it operates at a slow speed. Xie et al. (2020) [35] implemented a twofold LUT-based *tanh* function, but it comes at a high cost in hardware. Ref. [36] described a generic model for three types of nonlinear traditional activation functions based on a piecewise linear model. Pasca et al. (2018) [37] presented several floating-point architectures for the *sigmoid* and *tanh* functions and evaluated the impact of the activation function on both the area and latency of the RNN’s performance. They highlighted that the hardware area and latency can be significantly reduced if the *tanh*/*sigmoid* activation function is directly implemented. All of the implementations described above try to improve the accuracy between training and inference and aim to reduce hardware cost during inference. However, the activation function also influences the size of the neural network during training. Also, prior research has demonstrated that fixed activation functions based on linear segment approximation have a minimal impact on classification problems [38]. However, for the regression problems that we target, accuracy is a crucial requirement. The nonlinear characteristics of these fixed activation functions require an increased model size to achieve better training results. As a result, the hardware implementation in the inference phase for those functions is expensive.

In [11,12,14], it was shown that adaptive activation functions allow the networks to estimate a more accurate solution by training the activation function parameters during the training process. However, the hardware-based investigation of the AAF has received less attention than traditional activation functions. Ref. [39] implemented an adaptive ReLU activation function, but for an image classification problem. In this paper, we explore the accuracy and hardware efficiency of an AAF for regression in detail. We list the most popular activation functions in Table 1 along with the segmented spline activation function presented here.

Others [40] have presented a different implementation of the segmented spline approach and discussed other potential applications, specifically those that make use of deep neural networks.

### 3.2. Adaptive Activation Functions

Adaptive activation functions are a unique class of activation functions whose parameters are trainable and can adapt their shape and amplitude to the target dataset. Consequently, AAFs have better learning capabilities than fixed activation functions as they greatly improve the convergence rate, as well as the solution accuracy [11,12]. The segmented spline activation function, which is an AAF proposed in a previous paper [26], is defined as:

(1) f(x)=(C[i+1]−C[i])((x+1)Δ−i)+C[i]

where *C* is a trainable coefficient array that has length *L*, Δ=(L−1)/2 is the inverse of the *x*-axis width of a spline segment, and *i* is the segment index, which can be calculated by:

(2) i(x)=floor((x+1)Δ)

**Figure 3:**
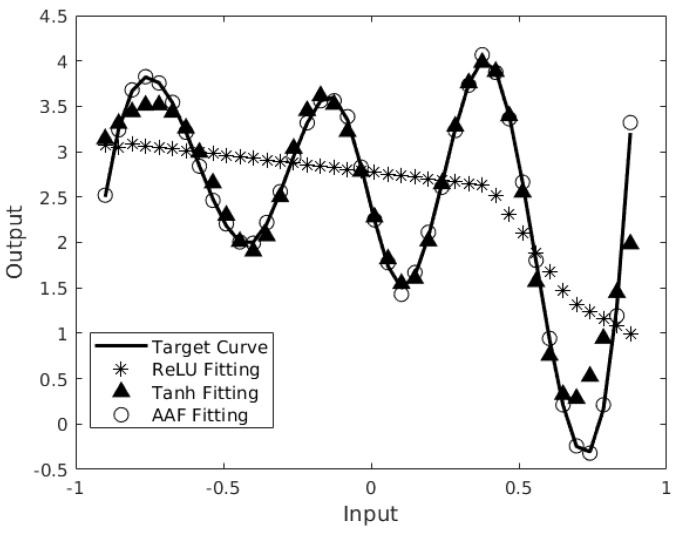
Segmented spline activation function vs. hyperbolic tangent activation function vs. ReLU activation function performance to mimic a nonlinear line.

This AAF directly implements the nonlinear function required by the neural network during training so that it achieves a better accuracy compared to other activation functions. In Figure 3, we evaluate the performance of a segmented spline activation-function-based on 9 neurons (represented by circles) against the performance of a hyperbolic tangent activation function and ReLU activation function both based on 9 neurons (represented by triangles and stars). All were used to model a nonlinear function (represented by a black line) in a double-precision floating point. Under the same settings for training, the results indicated that the AAF provided the best fit to the nonlinear function compared to the other traditional activation functions. The hyperbolic tangent function (tanh) has worse accuracy at the tops and bottoms of the target curve, while ReLU does not really follow the curve. This demonstrates that an adaptive activation function can be a more efficient solution for solving regression problems. This illustrates the effectiveness of the AAF with respect to training. The next step is to achieve an efficient hardware implementation of the AAF for inference so that the neural network can be implemented on the edge.

A previous paper [26] proved that the AAF-based SSCNN structure worked on DPD implemented in software and demonstrated its computational efficiency. To fully exploit the hardware efficiency of the segmented spline activation function, we designed the SSCNN hardware structures based on the previously presented mathematical algorithm and optimized it to achieve high-quality DPD for a PA while minimizing the amount of hardware required, as described in the next section.

## 4. Hardware Design and Implementation

While previous studies have explored various algorithmic approaches to optimize the structure of neural networks for DPD, few have implemented these strategies in hardware. Moreover, there is a lack of discussion of efficient implementations of the adaptive activation functions used in these networks. Given that nonlinear activation functions involve complex exponential and division computations, their hardware implementation is significantly more challenging than their linear counterparts. The challenge of fitting a neural network onto an edge device often comes down to trade-offs between accuracy, performance, and hardware cost due to resource limitations. In our study, we carefully designed the SSCNN at the circuit level based on its mathematical function. We also implemented various neural network structures to evaluate their DPD performance and compared the associated hardware costs among these with the SSCNN.

The activation function is an essential element in neural network-based applications, and it can effect the implementation of inference on edge devices. The same AAF is also critical during the training phase to provide the model with good prediction results. In our case, we first trained the neural networks using data collected from our test bench. The offline training contained two identical NN models: a predistorter and a postdistorter. We first sent the signal through the predistorter model for DPD, then, through the PA and collected the output. The output of the PA becomes the input for the postdistorter we are training, and the input to the PA is the target of training. This procedure iterates several times until the output PA has reached a linear standard. After each iteration, the predistorter’s parameters are updated by copying them from the trained model, the postdistorter. For the implementation, we implemented the final predistorter on the FPGA board and tested its output with the real PA in hardware to determine that we had achieved linear performance. The training and inference phases both make use of the segmented spline curve adaptive activation function.

### 4.1. SSCNN Model Analysis

Compared to the RVTDNN-based structures, the SSCNN structure does not need bias terms and eliminates nonlinear calculations, which save hardware resources in the implementation.

The configurable segmented spline activation function defined in Equation (1) can be achieved by the following steps:

**Step 1:**  Choose the coefficient array length *L*;
**Step 2:**  Calculate the inverse of the *x*-axis width of a single segment Δ=(L−1)/2;
**Step 3:**  Find the coefficient index i(x)=⌊(x+1)Δ⌋;
**Step 4:**  Access coefficients C[i+1] and C[i];
**Step 5:**  Achieve the activation function f(x)=(C[i+1]−C[i])((x+1)Δ−i)+C[i].

The resolution of the segmented spline activation function is determined by the first and second steps; it is flexible and can be adjusted during the training phase. The hardware improvements arise in the implementation of Step 3 to Step 5. These improvements are described in the remainder of this section. The coefficient index *i* calculated in Step 3 falls in the range between 1 and *L*, which is always larger than zero. Comparing the index *i* calculation function (Equation (2)) to the second multiplicand in Step 5, which is ((x+1)Δ−i), we noticed that the second multiplicand is actually the fractional part of (x+1)Δ, while the index *i* is the integral part of it. Based on this observation, we can split the integer and fractional part of the output of (x+1)Δ to efficiently achieve the activation function. Another thing to note is that the multiplication between (x+1) and Δ can be implemented as a shift operation if we choose the segment length *L* wisely.

### 4.2. SSC Layer Structure

**Figure 4:**
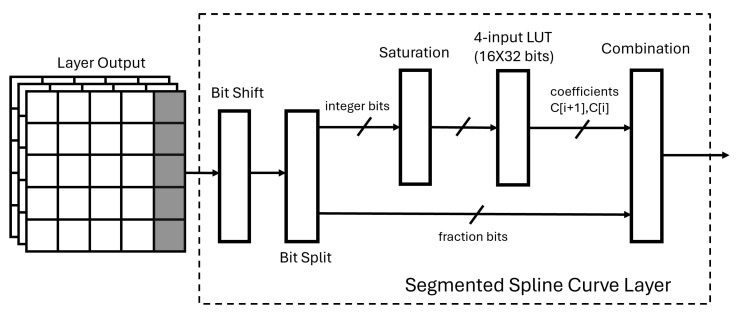
Segmented spline curve layer structure.

The only hidden layer of the SSCNN is the segmented spline curve (SSC) layer. Based on the mathematical analysis in the previous section, the segmented spline curve layer design can be divided into several small blocks. The block diagram of the SSC layer architecture is shown in Figure 4. The output of the previous layer is presented as the input matrix. The bit-shift block is used to multiply the input matrix by Δ defined in Equation (1). During the training phase, we chose the coefficient array length *L* to be a power of 2 plus 1 so that the Δ value will become a power of 2, and the multiplication can, therefore, be achieved by bit shifting. After shifting, a bit-split block is used for parallel processing of Step 3 and Step 5 from Section 4.1. The bits of the integer part from the shifting block are used as the address for the LUT-based distributed memory which contains the 32-bit-wide coefficients. As the LUT-based distributed memory size is limited by the segment length *L*, which was decided during the training phase, the integral part of (x+1)Δ should not exceed the memory address. Thus, those integers are truncated in the saturation block to prevent memory access overflow. For example, in our case, we chose the coefficient array length *L* to be 9, which means the integer bits after the saturation block are 4 bits to access the memory address between 0 and $2^{4}$. After saturation, the trainable coefficients in the memory are read while the bits of the fraction are sent to the combination block for calculation. Finally, the combination block combines all the parts needed to achieve the spline activation function. The output of the segmented spline cure layer will be passed to the output layer of the SSCNN shown in Figure 2.

### 4.3. Saturation

To prevent memory access overflow, the integer part from the bit-split block needs to fall into the range from 1 to *L*. The saturation process divides the input into three cases: Case 1: when the input is larger than or equal to the segment length *L*, it turns the output value into L−1; Case 2: when the input is smaller than 1, it outputs the value 1; Case 3: when the input is between 1 and *L*, it outputs the original value. Meanwhile, by adding this saturation step, the design improves the hardware efficiency by saving unnecessary decoding processes for LUT-based memory access as the integer after saturation is the address for the memory. For other LUT-based nonlinear activation functions, the integer part cannot directly be used to access the memory, so the decoding process is necessary for those functions, and the hardware cost consequently increases.

### 4.4. Systolic Processing

**Figure 5:**
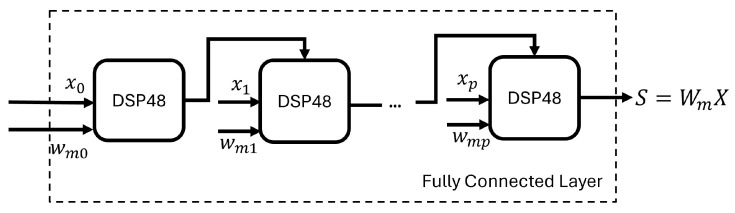
Systolic processing for fully connected layer. $x_{p}$ is the input vector of the layer; $w_{mp}$ is the weight vector; the output of each DSP48 slice is one of the addends for the next slice.

In addition to the SSC layer, the other layers (input layer and output layer) only include linear operations (weighted multiplications and additions). To process the data efficiently, the neuron structure in these other layers makes use of the DSP48 slices available in the FPGA fabric. Each neuron in fully connected layers needs to sum all output from the previous layer after it is multiplied by the weights. If the design processes all the multiplications in parallel and then adds the results, it will introduce idle time for the adders while increasing the delay between the multipliers and the adders. The optimal solution for this issue is to apply systolic processing for each layer. Figure 5 shows that, in a layer, the inputs are multiplied by the corresponding weights and accumulated in sequence to be fed into the activation function block efficiently.

### 4.5. Implementation

As mentioned earlier, many fixed-activation-function-based neural network structures have been proposed and optimized in previous research, but few AAFs have been implemented in hardware, so data concerning their implementation efficiency are lacking. In this research, we implemented five different network architectures with two different activation functions in the FPGA hardware: the hyperbolic-tangent-activation-function-based RVTDNN and ARVTDNN structures, two hyperbolic-tangent-activation-function-based fully connected deep neural networks, and the segmented-spline-activation-function-based SSCNN structure. The purpose of these implementations is to compare different activation-function-based neural networks on their DPD performance and their hardware efficiency.

#### Parameters

To ensure a fair comparison, we standardized the architecture of all shallow neural networks (RVTDNN, ARVTDNN, and SSCNN) to consist of one input layer, one hidden layer, and one output layer. In contrast, the two deep neural networks featured four to five layers, comprising one input layer, one output layer, and two to three fully connected hidden layers. This approach aims to evaluate diverse neural network structures within a similar framework. Specifically, the number of neurons in the input layer matches that of the first hidden layer. In all shallow neural networks, the number of hidden layer neurons was fixed at 9. Additionally, the ARVTDNN incorporated a first-order envelope term. For the SSCNN, we established a segment length *L* equal to 9, chosen because L−1 corresponds to a power of 2. For the deep neural networks, we configured the count of neurons in the hidden layers to be (9, 4) and (9, 4, 4) for the two respective networks. Across all structural variations, the output layer consistently comprised 2 neurons. In each of the neural networks, we established a memory depth of 2. The hyperbolic tangent activation function was applied in all hidden layers of the RVTDNN, ARVTDNN, and DNNs.

As the goal of training was to obtain an inverse PA model, in each DPD training iteration, the input of the PA would be the training target, and the output of the PA model would be fed into the next DPD training iteration. When the minimum error or a preset number of DPD training iterations was reached, the trained coefficients would be stored for DPD implementation. The whole training procedure finished in 2 DPD training iterations. Both the training and inference data contained 25,600 samples collected from the experimental setup. For the inference phase, all data are presented based on a 32-bit word length fixed point with 1 sign bit and 26 fraction bits. All fully connected layers in these five neural networks were implemented in the same systolic structure mentioned in Section 4.4 for a fair comparison. TO implement the hyperbolic tangent activation function, a 32-bit width by 300-unit LUT was used.

## 5. Results and Discussion

### 5.1. Experimental Setup

We conducted a performance assessment of digital pre-distortion using five distinct neural networks (discussed in Section 2) on the AMD/Xilinx RFSoC ZCU111 FPGA, San Jose, CA, USA board [41]. This board is equipped with the AMD/Xilinx Zynq UltraScale+ RFSoC ZCU28DR device, San Jose, CA USA, which offers high direct RF sampling rates. It utilizes 8 12-bit ADCs with a maximum rate of 4.096 GSPS and 8 14-bit DACs with a maximum rate of 6.554 GSPS. The programmable logic fabric of the device comprises 4272 DSP slices, 930 K system logic cells, and 60.5 Mb of memory. In addition to the FPGA fabric, this system-on-chip (SoC) also incorporates a full Arm processing subsystem, forming a heterogeneous compute architecture. The device is equipped with 4 GB of Programmable Logic (PL) DDR4 memory and 4 GB of Processing System (PS) DDR4 memory. We connected the AMD XM500 RFMC balun transformer, San Jose, CA, USA add-on card to the device via its expansion connectors to establish a complete software-defined radio (SDR) chain.

**Figure 6:**
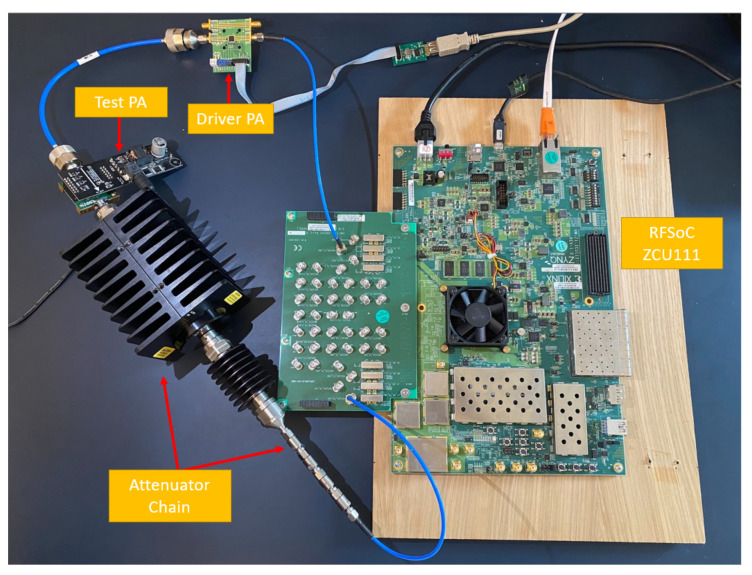
Experimental setup AMD/Xilinx RFSoC ZCU111 board, XM500 RFMC balun board, NXP AFSC5G37D37 Doherty PA (test PA), PA NXP BGA7210 (driver PA), (a 40 dB attenuator chain).

The experiment test bench consisted of an AMD/Xilinx RFSoC ZCU111 board with an NXP AFSC5G37D37 Doherty PA, Eindhoven, The Netherlands and a linear driver stage PA NXP BGA7210, Eindhoven, The Netherlands. The transmit signal sent from the DAC goes through the driver PA and the test PA, then goes back to the ADC through a 40 dB attenuator chain, as shown in Figure 6. A 40 MHz bandwidth OFDM waveform, commonly used in the LTE and 4G/5G standards, is transmitted at a center frequency of 3.8 GHz. The transmit signal sent from the host computer goes through the Processor System (PS) side DDR4 memory and then is stored in the external DDR4 on the Programmable Logic (PL) side. On the PL side, the DPD models with the synchronization block are applied to predistort the signal and then store the preprocessed signal back in the memory. The RF frontend DAC will transmit the signal by reading the DDR4 memory repeatedly. After the signal passes through the power amplifiers, the PAs’ outputs will be written back to the PS and read by the host computer. The offline training for DPD was carried out with the Deep Learning Toolbox in MATLAB R2023a. DPD IPs were constructed in Simulink and, then, translated to Verilog using the MATLAB HDL Coder. The entire synthesis and implementation process was completed in *Vivado 2020.1*.

### 5.2. DPD Performance

In power amplifier linearization, three key performance metrics are typically used: Normalized Mean-Squared Error (NMSE), Adjacent Channel Power Ratio (ACPR), and Error Vector Magnitude (EVM). The NMSE quantifies the in-band distortion at the PA’s output. The ACPR, on the other hand, quantifies the relative power that leaks into neighboring frequency channels due to the PA’s nonlinearity, which helps evaluate the level of interference a signal from that transmitter may cause to other signals in adjacent frequency bands. Lastly, the EVM is used to measure the discrepancy between the ideal and actual signal, providing an assessment of the quality of the transmitted signal.

Table 2 presents the DPD performance across various neural network model structures. The number in each model name represents the number of neurons in the hidden layer(s). Among the similar architectures, RVTDNN and ARVTDNN, the latter requires more coefficients due to its inclusion of additional linear inputs. However, the ARVTDNN exhibits slightly superior performance compared to the RVTDNN. In comparison to the ARVTDNN, the DNN provides no improvement by introducing one layer with a very small number of neurons, but requires 30 more coefficients. The DNN with three hidden layers increased the performance compared to the ARVTDNN at the cost of 50 extra coefficients. The SSCNN, on the other hand, attained a performance level comparable to the ARVTDNN and three-hidden-layer DNN models while utilizing a similar number of coefficients as the RVTDNN. Compared to the PA output without DPD, the RVTDNN with merely nine hidden neurons exhibited the poorest performance, while the SSCNN with an equivalent number of hidden neurons delivered the best performance. It should be noticed that, for a fair comparison of hardware utilization later, these neural network models were intentionally set with similar parameters.

**Figure 7:**
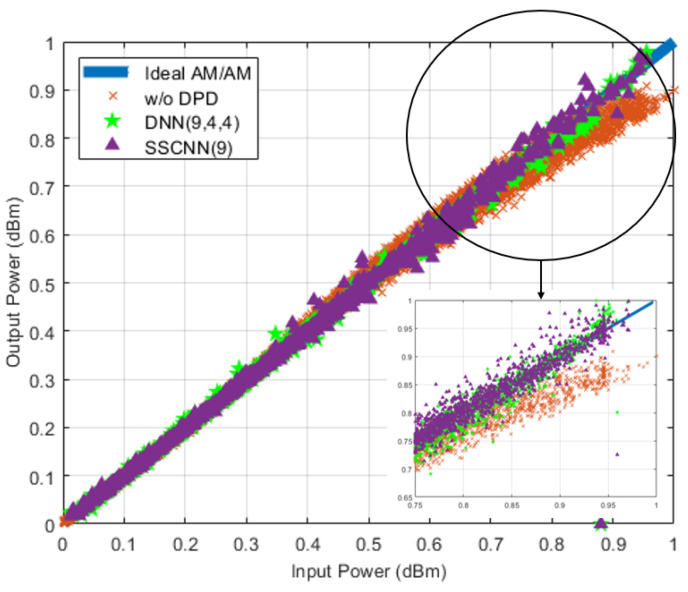
AM/AM curve. Two- hidden-layer-based DNNs vs. SSCNN in-band performance. Zooming into the nonlinear range shows the PA’s performance after the DNN(9, 4, 4) (green star) and SSCNN(9) (purple triangle) are close to the ideal PA’s AM/AM curve (blue line) compared to the PA’s performance w/o DPD (orange cross).

In Figure 7, we show the AM/AM curves based on the SSCNN and three-hidden-layer DNN. The AM/AM curve represents how the amplitude of the output signal changes as a function of the amplitude of the input signal. The in-band linearization performance of both neural network-based DPDs was compared with the PA’s performance without DPD. The blue line signifies the ideal linear output of the PA, while the red “x” line depicts the actual behavior of the PA without DPD. The green star and purple triangles represent the performance of the three-hidden-layer DNN-based DPD and the SSCNN-based DPD. It shows that the two neural network-based DPDs successfully corrected the PA’s nonlinear behavior, as both the green star and purple triangles form a straight line close to the ideal output of the PA. The SSCNN performed comparable in-band linearization as the three-hidden-layer DNN. In the case of DPD, if the training signal covers the intended range of operation for the power amplifier hardware, then different neural networks can be trained to accurately react to unforeseen inputs. This implies that the NN will cover the frequency of operation, power level, signal bandwidth, and signal modulation scheme. A major performance benefit that the SSCNN offers is its ability to represent this range of operation with a much smaller number of neurons. The smaller number of neurons will in general result in the SSCNN having faster training and operation speed than a DNN.

**Figure 8:**
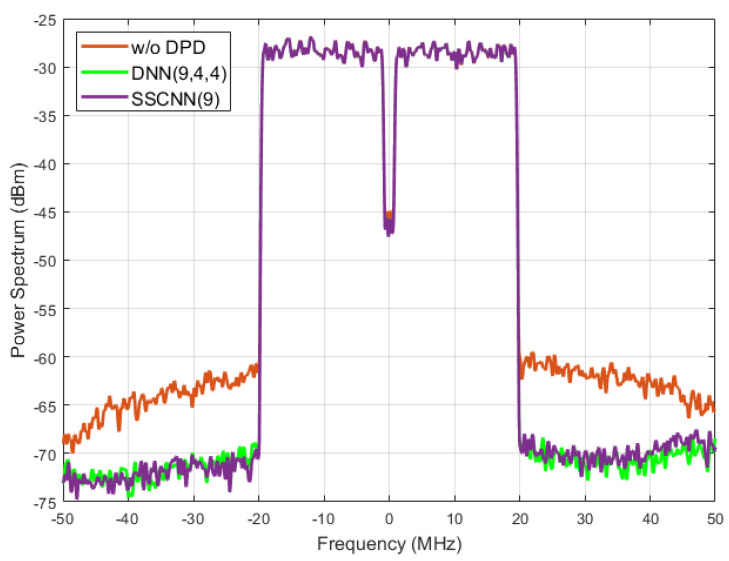
Spectrum performance improvement. At least 8 dBm power drop between the original PA output (orange line) and the two neural network-based DPD outputs (green and purple lines).

**Figure 9:**
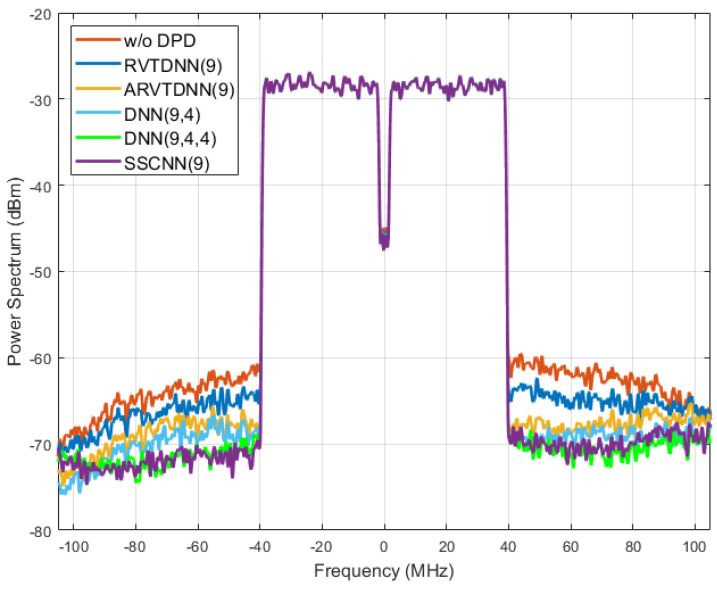
Spectrum performance of five neural networks. The RVTDNN (9) reduces by only 4 dBm while both the DNN (9, 4, 4) and SSCNN (9) reduce by at least 8 dBm.

Figure 8 shows the two DPD’s out-of-band linearization performance. The power spectrum refers to the distribution of power into frequency components composing that signal. Ideally, the power should be focused on the transmitted signal’s baseband. The PA’s nonlinear performance will lead to an increase in power beyond the baseband range and leak power to a neighboring channel to cause interference. As the orange line indicates here, outside of the bandwidth of the transmitted signal, the power was above −70 dBm. This line indicates the signal transmitted after the PA, without DPD. The green and purple lines, showing the performances of the three-hidden-layer DNN and SSCNN, represent that the transmitted signal’s shoulders are notably reduced compared to the transmitted signal without predistortion. This reduction signifies a decrease in the risk of the PA-transmitted signal interfering with adjacent channels when employing NN-based DPD. Once again, the SSCNN and the DNN with three hidden layers produced a similar degree of out-of-band linearization. Figure 9 presents all five models’ spectrum performance.

From an effective DPD perspective, these results show that, with a fixed activation function, the DNN is not always the best solution as a similar structure, comparing the DNN (9, 4, 4) with SSCNN (9). The DNN requires the most coefficients while only offering limited performance improvements. On the other hand, comparing the SSCNN (9) with the DNN (9, 4) shows that, with an adaptive activation function, the model can significantly reduce the depth of neural networks while having great accuracy. This emphasizes the importance of developing a hardware-efficient adaptive-function-based model for neural network implementation on edge devices.

### 5.3. Resource Utilization

Table 3 shows the resource utilization for the different DPD models explored in this paper. The first thing to note is that the deep neural network required the most hardware resources. Combined with its performance (see Table 2), we found that the DNN had limited efficiency for DPD implementation on edge devices not only because of its expensive computational cost, but also because of its slow speed. The delay that caused the slow speed was not only from the increased layer number, but also from the latency to access the coefficients stored in the BRAM for the nonlinear activation functions. On the other hand, the SSCNN had a similar number of coefficients as the RVTDNN, but the best performance, as shown in Table 2, while using similar hardware resources needed for the RVTDNN. Compared to the RVTDNN’s cost, the SSCNN does not require any BRAMs. This is due to the fact that we replaced the complicated nonlinear activation functions with an optimized adaptive activation function, which significantly reduced the memory cost. Specifically, the SSCNN(9) used 32% less slice LUTs, 42% less FFs, 100% less BRAMs, and 41% less DSPs compared to the DNN (9, 4, 4). Meanwhile, by eliminating the latency to access the BRAM and using optimized systolic processing, the speed of the SSCNN increased by 117% compared to the DNN models. The RVTDNN cost less resources than the ARVTDNN, but sacrificedthe accuracy. The ARVTDNN, which obtained the second-best performance among the shallow neural networks, had comparable performance to the DNN while still costing less than the DNN. Among all the different models, the SSCNN provided the most efficient hardware solution as it had the fastest speed and the least hardware cost while reaching a similar level of DPD performance compared to the DNN models.

## 6. Conclusions and Future Work

Applying neural networks on the edge where they can be used to directly process transmitted and received signals is a growing area in FPGA implementations for wireless communications. Efficient neural networks that are hardware friendly can achieve both high performance and low hardware usage, freeing the FPGA to implement other functions. Digital predistortion is a critical technique to remove nonlinear distortion from wireless signals. FPGAs offer an ideal platform to implement the predistorter in these real-time communication systems. Deep neural network-based predistorters have been proposed; however, they typically require large amounts of hardware resources to be realized. In this work, an implementation of an adaptive-activation-function-based shallow neural network with a very small footprint in the FPGA fabric compared to other fixed-activation-function-based approaches has been detailed. The final implementation demonstrated the substantial reduction of the resources required for the SSCNN compared to previous techniques, as well as the comparable performance from a communications perspective. This reduction in required hardware means more programmable logic resources are available for additional functions such as signal interpolation, filtering, error correction, modulation, and encoding, such as non-orthogonal multiple access (NOMA) for 6G. In future research, we plan to build an online NN-based DPD system that includes both training and inference on the edge. As compared with 5G, 6G is expected to provide ten-times lower latency, which requires a faster and more adaptive DPD real-time system for more complicated dynamic scenarios.

## Figures and Tables

**Table 1:** Commonly used activation functions and segmented spline activation function.

Activation Function	Definition
ReLU	f(x)=max(0, x)
Sigmoid	$f\left(x\right)=\frac{1}{1+e^{-x}}$
Hyperbolic Tangent	$f\left(x\right)=\frac{e^{x}-e^{-x}}{e^{x}+e^{-x}}$
Segmented Spline	f(x)=(C[i+1]−C[i])((x+1)Δ−i)+C[i]

**Table 2:** Linearity performance for different neural networks.

Model	# of Coefficients	EVM (%)	ACPR (dBm)	NMSE (dB)
No DPD	-	4.0725	−35.487	−27.803
RVTDNN (9)	83	2.7758	−37.752	−31.132
ARVTDNN (9)	110	2.4284	−40.748	−32.295
DNN (9, 4)	140	2.4310	−40.138	−32.284
DNN (9, 4, 4)	160	2.2545	−41.650	−32.939
SSCNN (9)	85	2.3340	−41.886	−32.653

**Table 3:** Resource utilization for different neural networks.

Model	Slice LUTs	FFs	BRAMs	DSPs	Clock Speed (MHz)
RVTDNN (9)	6123 (1.44%)	7880 (0.93%)	4.5 (0.42%)	90 (2.11%)	118.73
ARVTDNN (9)	7289 (1.71%)	10,935 (1.29%)	4.5 (0.42%)	117 (2.74%)	116.71
DNN (9, 4)	9355 (2.2%)	12,425 (1.46%)	6 (0.56%)	152 (3.56%)	102.25
DNN (9, 4, 4)	12,080 (2.84%)	14,072 (1.65%)	8 (0.74%)	184 (4.31%)	100.68
SSCNN(9)	8258 (1.94%)	8084 (0.95%)	0 (0%)	108 (2.53%)	221.12
